# Challenges in the Management of Infectious Complications in Neutropenic Cancer Patients

**DOI:** 10.3390/microorganisms12112315

**Published:** 2024-11-14

**Authors:** Carlota Gudiol

**Affiliations:** 1 Infectious Diseases Department, Bellvitge University Hospital, Bellvitge Biomedical Research Institute (IDIBELL), 08907 Barcelona, Spain; cgudiol@bellvitgehospital.cat; 2 Faculty of Medicine, Bellvitge Campus, University of Barcelona, 08907 Barcelona, Spain; 3 Institut Català d’Oncologia (ICO)–Hospital Duran i Reynals, Bellvitge Biomedical Research Institute (IDIBELL), 08907 Barcelona, Spain; 4 Centro de Investigación Biomédica en Red de Enfermedades Infecciosas (CIBERINFEC), Instituto de Salud Carlos III, 28029 Madrid, Spain

## 1. Introduction

Febrile neutropenia is a very common complication in cancer patients, especially in hematologic patients and hematopoietic stem cell transplant (HSCT) recipients, and is associated with increased morbidity and mortality [1]. In these patients, bacterial and fungal infections are frequent and have become one of the most important challenging clinical situations in this setting. On the one hand, the current emergence of multidrug-resistant Gram-negative bacilli is worrisome, since inadequate initial empirical antibiotic therapy puts these patients at high risk for poor outcomes [2,3,4]. On the other hand, invasive fungal diseases (IFDs) are a significant concern in hematological patients, since breakthrough IFD in the setting of broad-spectrum antifungal prophylaxis make diagnostic and therapeutic management difficult [5,6,7]. In this collection of articles, the authors explore various aspects of infections in this vulnerable population, highlighting significant findings and discussing potential strategies for improvement.

## 2. Overview of Published Articles

### 2.1. Pseudomonas aeruginosa Bloodstream Infections in Neutropenic Patients

This article is a comprehensive multicenter retrospective study that investigates the prevalence and impact of septic shock among neutropenic patients with *Pseudomonas aeruginosa* bloodstream infections (PABSIs). This large study examines 1213 episodes of PABSI in onco-hematological neutropenic patients, finding that 33% presented with septic shock. The study identifies key risk factors for septic shock, including solid tumors, high Multinational Association for Supportive Care in Cancer (MASCC) scores, pneumonia, and multidrug-resistant *P. aeruginosa* infections. Inadequate empirical antibiotic therapy and poorer outcomes were more common in these patients. The research emphasizes the need for tailored management strategies to improve survival rates.

### 2.2. Efficacy of Ceftazidime–Avibactam for Klebsiella pneumoniae Bloodstream Infections

This prospective multicenter observational study evaluates the effectiveness of ceftazidime–avibactam (CA) for the treatment of *Klebsiella pneumoniae* carbapenemase-producing Enterobacterales bacteremia (KPC-PEB) in high-risk neutropenic patients. The study compares CA-treated patients with those treated with other antibiotics and those with ESBL-producing Enterobacterales. CA-treated patients showed higher clinical response rates and lower overall and infection-related mortality, suggesting CA as a preferred therapeutic option for KPC-PEB in this high-risk population.

### 2.3. Invasive Fungal Diseases in Hematological Malignancy Patients

This narrative review and retrospective study focus on the incidence, diagnosis, and treatment of breakthrough invasive fungal diseases (IFDs) in patients with acute myeloid leukemia and myelodysplastic syndrome undergoing chemotherapy and allogeneic hematopoietic stem cell transplantation. The study highlights the challenges in managing IFDs, noting the incidence of breakthrough infections despite prophylaxis and discussing the need for improved diagnostic techniques and new antifungal treatments.

### 2.4. Invasive Fungal Infections in Pediatric Acute Leukemia

This retrospective study analyzes febrile neutropenic episodes in children with acute leukemia, assessing factors associated with proven/probable IFDs. The study found that 18.3% of children developed IFDs, with higher rates in high-risk acute lymphocytic leukemia and acute myeloid leukemia. Risk factors such as underlying genetic syndromes and oral mucositis were identified, underscoring the need for close monitoring and preventive strategies in pediatric patients.

### 2.5. Utility of 18F-FDG-PET-CT in Febrile Neutropenia Management

This study evaluates the role of 18F-FDG-PET-CT in managing febrile neutropenia in patients with hematological malignancies. Compared to conventional imaging, 18F-FDG-PET-CT provided benefit in diagnosing infection sites, excluding infections, and guiding antimicrobial therapy adjustments. The findings, supported by a systematic review of the literature, suggest that 18F-FDG-PET-CT is useful in managing febrile neutropenia, particularly for diagnosing fungal infections and optimizing antimicrobial use, though further research is needed.

## 3. Conclusions

The collective findings from these five studies underscore the significant challenges and complexities in managing infections among neutropenic patients, particularly those with hematologic malignancies. These studies provide crucial insights into various aspects of infection management, highlighting both persistent challenges and potential strategies for improvement. Together, these studies highlight the critical need for the ongoing research and development of innovative diagnostic and therapeutic strategies to better manage infections in neutropenic patients. Improved risk stratification, timely and appropriate antimicrobial therapy, and advanced diagnostic techniques are essential components in enhancing patient care and outcomes in this high-risk population.
